# Acceptability and Feasibility of Single-Component Primary School Physical Activity Interventions to Inform the AS:Sk Project

**DOI:** 10.3390/children5120171

**Published:** 2018-12-17

**Authors:** Sarah L. Taylor, Robert J. Noonan, Zoe R. Knowles, Bronagh McGrane, Whitney B. Curry, Stuart J. Fairclough

**Affiliations:** 1 Physical Activity and Health Research Group, Sport and Physical Activity Department, Edge Hill University, Ormskirk, Lancs L39 4QP, UK; robert.noonan@edgehill.ac.uk (R.J.N.); stuart.fairclough@edgehill.ac.uk (S.J.F.); 2 Physical Activity Exchange, Research Institute for Sport and Exercise Sciences, Liverpool John Moores University, Liverpool L3 2AT, UK; Z.R.Knowles@ljmu.ac.uk; 3 School of Arts Education & Movement Department, Dublin City University Institute of Education, St Patrick’s Campus, Dublin, Ireland; bronagh.mcgrane@dcu.ie; 4 Wellbeing and Public Health, Cornwall Council, Truro TR1 3AY, UK; whitney.curry@cornwall.gov.uk; 5 Department of Physical Education and Sports Science, University of Limerick, Limerick, Ireland

**Keywords:** acceptability, feasibility, intervention, physical activity, sedentary, accelerometry, children

## Abstract

Multi-component school-based interventions provide physical activity (PA) opportunities for children but are often difficult for schools to execute and may not be implemented as intended. The primary aim of this study was to explore the acceptability and feasibility of three brief single-component primary school PA interventions targeting 9–10-year-old children. The secondary aim was to examine the effectiveness of the interventions on increasing PA levels and reducing sedentary time. The single-component interventions included active classroom breaks (AB; 3 schools; *n* = 119 children) Born to Move (BTM) exercise videos (2 schools; *n* = 50 children), and playground supervisory staff training (2 schools; *n* = 56 children). Qualitative data from participating children (*n* = 211), class teachers (*n* = 6), and playground supervisory staff (*n* = 8) explored the experiences, acceptability, and feasibility of each intervention component. Accelerometers were worn by 225 children during the last week of implementation. Teachers reported that they were able to implement ABs daily, but BTM videos were more difficult to implement daily because of accessing sufficient space. Playground staff reported difficulties in implementing activities due to children’s age and competing responsibilities on the staffs’ time. Children reported that the ABs and BTM videos were enjoyable. During half hour time windows, including the ABs and BTM videos, children engaged in 4.8 min and 8.6 min of moderate to vigorous PA (MVPA) on average, respectively. ABs and BTM videos positively affected MVPA. ABs were feasible to implement; however, teachers faced some barriers in implementing the BTM videos. Feasibility of playground interventions may be dependent on staff responsibilities and age of the children.

## 1. Introduction

Physical activity (PA) can have beneficial effects on physical and psychological health in school-aged children [1,2,3]. On average, children aged 4–18 years engage in 30 min of moderate to vigorous physical activity (MVPA) daily, only half of the recommended 60 min MVPA per day [4,5]. Furthermore, sedentary behaviors are detrimental to many aspects of health, such as body composition, cardiorespiratory fitness, metabolic syndrome, and cardiovascular disease risk factors [6], and there is evidence that children’s sedentary time (ST) increases during the transition from primary/middle to secondary/high school [7]. Therefore, interventions aimed at improving PA levels and reducing ST are warranted to improve the health outcomes in children and young people.

Schools are key environments in which to promote PA participation among children because children and adolescents spend most waking weekday hours in school and there are many opportunities to promote PA within the school day [8,9]. However, children engage in both non-recreational (schoolwork) and recreational ST within school [9], which can account for 65% of the children’s time at school [10]. The Institute of Medicine’s committee on PA in the school environment recommends that more than half of the recommended 60 min of MVPA should be accomplished during school hours [9]. Whole-school approaches are advocated as a means of engineering a range of PA opportunities into the day, using a variety of strategies across different school settings [11]. For example, comprehensive school PA programming (CSPAP) comprises of five different components or points of intervention, including physical education (PE), PA during school, PA before and after school, staff involvement, and family and community involvement, thereby developing a school culture that is conducive to promoting lifelong PA [12]. Results from a meta-analysis published in 2015, indicated that as the number of CSPAP intervention components increases, the effect size associated with the change in daily PA also increases [13]. Action Schools! BC (AS!BC) is an ongoing example of an effective multi-component intervention that is consistent with the concept of a whole-school approach [14]. An important aspect of this intervention was that programs were customized based on the perceived needs of the schools and it included activities across six action zones of: School environment, scheduled PE, classroom action, family and community, extra-curricular, and school spirit [15]. The intervention led to schools providing approximately 10 extra minutes of PA per day [16].

Nonetheless, it has been reported that multi-component interventions are difficult to implement [17]. Subsequently, schools may not implement interventions as intended, which can result in a lack of success in positively effecting PA levels [17]. The use of a CSPAP approach and frameworks such as the theory of expanded, extended, and enhanced opportunities (TEO), designed to help interventions identify appropriate targets across different settings and contexts [18], can help to design more feasible interventions. Moreover, formative research is important to better understand the context-specific needs of different schools. Before implementing complex multi-component PA interventions, investigating the suitability of individual components may first be warranted to understand the components feasibility and acceptability in specific school contexts. This reflects the UK Medical Research Council’s (MRC) best practice for implementing complex interventions, which recommends a phased approach with a feasibility stage testing for acceptability [19].

The increasing demands placed upon teachers and school staff to cover curriculum content and achieve academic targets should be considered alongside the feasibility and acceptability aspects. Teachers recognize the benefits of PA in improving learning, but they have reported lack of time as the key barrier to implementing daily PA [20,21]. School-based PA interventions need to be effective at increasing PA levels, but they also need to be feasible for teachers to implement within a time constrained school day. Acceptability and feasibility of interventions should therefore be examined with the experiences and views of key agents, such as teachers and children, using qualitative methods [22,23]. 

Formative research conducted prior to the implantation of multi-component school-based interventions to inform content is limited. Process evaluation research can uncover barriers and facilitators towards implementation, but this occurs post-implementation [24]. Owing to the reported difficulties associated with the implementation of multi-component interventions within the school setting [17], there is a need for intervention strategies which are feasible for teachers to implement. Before PA strategies are combined and implemented as multi-component interventions, formative research to explore the target audiences’ perceived feasibility and acceptability of implementing single-component school-based PA strategies is warranted. If single-component strategies are perceived to be feasible and acceptable, or if recommendations are made to increase the strategies feasibility and acceptability, then once combined within multi-component interventions, implementation may occur as intended. Furthermore, whilst not advocating for the implementation of single-component interventions overall, the value of these interventions is not to be undermined. For example, research has indicated that school-based PA interventions have been shown to have a significant effect on cardiorespiratory endurance in primary school-aged children, and the intervention component number does not modify this significant effect [25].

The primary aim of this study was to explore the acceptability and feasibility of three brief single-component primary school PA interventions. The secondary aim was to examine the effectiveness of the interventions on increasing PA levels and reducing ST. The findings will be used to inform a multi-component intervention which will be implemented as part of the Active Schools: Skelmersdale (AS:Sk) project, with a view to the single-component interventions being integrated as part of this project.

## 2. Materials and Methods

### 2.1. Participants and Recruitment

This study was the second phase of the AS:Sk project, and it took place between January and March 2017. Phase one included the collection of 7-day accelerometry data and the exploration of child and school-level predictors of segmented school day PA and ST as described in Taylor et al., 2017 [26]. Phase three included the evaluation of a multi-component school-based PA clustered randomized controlled trial as described in Taylor et al., 2018 [27]. Seven primary schools within Skelmersdale, a low income town, within West Lancashire, UK participated in the project [28]. All the participating schools took part in this second phase without the use of a control group due to the focus on feasibility and gaining an understanding of whether something can be done, should we proceed with it, and if so, how [29]. Once ethical approval from the University Research Ethics Committee was granted (ref #SPA-REC-2015-183), the schools received the relevant paperwork to inform each Year 5 child (*n* = 237, age 9–10 years) about the study. Passive (“opt-out”) parental consent was obtained in six of the schools, where parents/carers only returned a completed form if they did not want their child to participate. One school chose to use active parental consent, where parents/carers returned a completed form if they did want their child to participate. Children completed informed assent forms prior to data collection. This process resulted in 225 participating children (109 girls, 95% recruitment rate).

### 2.2. Intervention Components

Participating schools were invited to project meetings to review phase 1 (7-day accelerometry data) of the AS:Sk project and to discuss phase 2 interventions. A list of six potential interventions for phase 2 was presented to the schools from which they were given the opportunity to select one that aligned best to the areas of their school day that they felt were most in need of intervention. All the intervention components were selected or designed based on effective interventions within the literature, and they were to have no/limited cost to the project or to the schools to implement, and existing partnerships were explored for available opportunities. The project team then discussed potential feasibility of the approaches before proposing the six intervention components to the participating schools. These were: Activity promoting pedagogical practices and training for PE, playground supervisory staff training for PA with activity ideas, changes to recess policies or rules (e.g., increase in time available), active breaks (ABs), daily walking or running club, and daily Born To Move videos (BTM; http://www.lesmills.com/borntomove).

#### 2.2.1. Active Breaks Intervention

Three schools chose to implement ABs (*n* = 119 children). Twenty-three activity cards were created with pictures on the front demonstrating the activity and instructions on the back. Each activity card was designed to last for 30 s with ABs recommended to last for five minutes in total. This time period was chosen to cause minimal disruption to class time and it was used in a pilot of a primary school AB programme (ACTI-BREAK) as outlined by Watson et al., 2017 [30]. The ACTI-BREAK pilot study, in which participating schools were instructed to implement five-minute ABs three times per day, did not have an effect on PA levels [31]. It is possible that this pilot study was not successful at positively impacting PA levels due to issues of fidelity in delivery or the activities lacking sufficient intensity [31]. When designing the activities for the ABs in the current study, it was considered that they should be suitable for use within the limitations of typical classroom space and of a sufficient intensity to ensure engagement in PA occurred. A recent review of classroom-based PA interventions has reported small increases in PA through the use of ABs, as well as positive impacts on academic outcomes [32]. In the current study, teachers could perform an AB for shorter or longer periods if they wished and they were asked to implement at least one AB per day.

#### 2.2.2. Born to Move Videos Intervention

Two schools chose to implement daily BTM structured exercise videos (*n* = 50 children). It was recommended that videos were used as a break to classroom learning. Videos were 10 min in duration and required hall/gym space with a projector screen. Videos included age-appropriate motor skills within a fluent and structured routine set to contemporary music designed to improve health-related and skill-related fitness. Each video included a Les Mills instructor who guided children through the moves with clear instructions and demonstrations, thus no input was required from the class teacher. A recent evaluation of the BTM pilot programme concluded that live 30-min BTM lessons delivered by a trained instructor engaged children in significantly more MPA than during regular physical education (PE) lessons [33]. This was the first evaluation of the 10-min BTM videos, which were more appropriate for use to break up the school day ST due to the shorter duration. Moreover, the videos also do not require a trained instructor to implement as the children are directed by the video content.

#### 2.2.3. Playground Intervention

Two schools chose to implement playground active games, including a training session for playground supervisory staff (*n* = 56 children). Teachers who have previously engaged in PA based professional development have reported it to be highly valuable [34]. The training session lasted for 60–90 min and it was delivered by the lead author and a PE professional development specialist from the West Lancashire Sport Partnership, which delivers PA programs in the participating schools. Training covered the importance of PA for health, wellbeing, and learning, and ideas for engaging less active children on the playground with activity. These ideas included the use of a student leadership program with older children acting as play leaders to initiate activity with younger children, and also teacher engagement with activities and teacher reinforcement with praise and encouragement for children. Schools were provided with booklets of active games which required little or no equipment and were easy to set up. Playground supervisory staff were asked to implement active games daily and although they were informed that the project was targeting children aged 9–10 years, they were asked not to exclusively target these children on the playground. Given that the other intervention approaches of this study exclusively targeted 9–10-year-old children, it was considered that the playground supervisory staff could also do this and exclusively target 9–10-year-old children when implementing the playground games. However, the decision to not ask playground supervisory staff to do this was based on pragmatic reasons. Playground breaks include children of all ages. Asking a supervisor to tell other children who may wish to participate and be physically active that they cannot take part because they were too young or too old was deemed unfair to implement.

### 2.3. Measures

The study employed a mixed methods design, generating both qualitative and quantitative data. These separate data sources were collected and analyzed independently, but later pooled together for complementary purposes. Adopting such an approach enabled the study’s aims to be achieved.

#### 2.3.1. Qualitative Data

All children who were present on the day of data collection took part in a group interview conducted on the same day (*n* = 32 group interviews; *n* = 111 children AB intervention; *n* = 48 children BTM intervention; *n* = 52 children playground intervention) to explore the children’s experiences of the interventions. Group interviews were conducted with all available children (rather than a sub-sample) to assess acceptability across every school. Additionally, group interviews were deemed more appropriate than focus groups to assess acceptability. Within group interviews, opinions from individuals are collected within a group setting and conversation is largely dictated by the interviewer directed to each individual in the group as described in Coe et al., 2017 [35]. This is unlike a focus group setting, where the researcher facilitates or moderates a group discussion between participants and not between the researcher and the participants [36]. To gain an understanding of the intervention acceptability which had been implemented, it was important that the researcher could dictate questions to achieve this. Additionally, group interviews allow for individual accounts to be collected which are suited to feasibility outcomes. Conversely, within focus groups attention is paid to group consensus. Conducting individual interviews to collect individual accounts is time demanding; therefore, group interviews are most suitable. To ensure that all the children participated, questions were directed at each individual child to ensure that all participating children contributed to answering the questions equally.

Group interview size was between five and seven children with allocations pre-determined by teachers, as this was most convenient. Group interviews were conducted by the lead author who had previous experience of collecting qualitative data. Two research assistants were recruited to also conduct the group interviews and received basic training prior to this. A semi-structured format using open-ended questions ensured consistency across the interviews for each intervention. Example questions from the interview guide included, “what did you like about the new activities?”; “was there anything you didn’t like about the activities?”; “how would you feel if your teacher decided to stop doing the activities with your class?” The group interviews took place in a quiet area in the school where participants could be overlooked but not overheard and lasted 5–19 (mean = 10) min. Interviews were recorded using a digital recorder and transcribed verbatim resulting in 392 pages of raw transcription data, Arial font, size 12, double spaced.

Six class teachers (*n* = 3 AB intervention teachers; *n* = 3 BTM intervention teachers) and eight playground supervisory staff who were responsible for implementing the intervention components were interviewed by the first author. Interviews were conducted as four individual interviews and three group interviews. Group interviews were arranged for convenience, particularly for playground staff who were not full-time members of staff. All participants were given the opportunity to respond to each question in turn regardless of whether it was in a group or individual interview, and discussion within groups was not permitted. Semi-structured interview guides with open-ended questions were used. Example questions from the interview guide included, “how much planning was/is required to implement the intervention?”; “were there any barriers which prevented you from implementing the intervention on certain days?”; “do you think you would be able to sustain the intervention across a full school year”; “is there anything you would need to be able to do so?.” The interviews took place in a quiet, private area of the school at a convenient time and lasted 6–22 (mean = 12.4) min. Teacher interview data consisted of 65 transcript pages, Arial font, size 12, double spaced.

#### 2.3.2. Quantitative Data

During the final week of the four-week intervention period, the children wore an accelerometer on their non-dominant wrist for seven consecutive days (24 h·day^−1^), removing it only for water-based activities. Teachers also completed a recording sheet each day during the four-week intervention period (for the AB and BTM interventions only). Teachers stated the start and end times of the intervention period each day, so that the accelerometer data could be examined for these time periods. For the playground intervention, active games did not have a definite time period so playground staff were not asked to report any implementation times. Wrist-worn accelerometry can promote better wear compliance compared to hip-worn devices with children [37]. Limited availability of accelerometers meant that a combination of ActiGraph GT9X (AG; Pensacola, FL, USA) and GENEActiv (GA; Activinsights, Cambs, UK) devices were used (AG *n* = 93, GA *n* = 132). Consistency of accelerometer devices within schools was possible; however, consistency of accelerometer devices within the intervention type was not possible. Agreement between the GA and AG devices was investigated by Rowlands et al [38], who reported that AG accelerations were 9–11% lower than GA for the same activities, but that the time spent in ST and light PA (LPA) thresholds was statistically equivalent [38]. Furthermore, although it was not within the 10% equivalence zone, the agreement between the devices for MVPA was high [38]. As the primary focus of this study was on intervention acceptability and feasibility, rather than on activity levels, we decided to combine the AG and GA data, while at the same time acknowledging the associated equivalency issues. Both devices were initialized to record raw accelerations at a frequency of 30 Hz. After 7 days of wear, the data was downloaded to a format which facilitated raw data processing (AG; ActiLife v6.11.8 saved as GT3X files and converted to CSV format; GA; GA v2.2 software saved as binary files). 

Files were processed in R (http://cran.r-project.org) using the package GGIR (version 1.5–17). GGIR converted the raw triaxial accelerometer signals into one omnidirectional measure of acceleration termed the Euclidean norm minus one (ENMO) as described in van Hees et al., 2013, 2014 [39,40]. ENMO values were averaged per 1 s epoch over each of the seven monitored days as in Fairclough et al., 2016 [37]. Non-wear time was determined using the methodology used in previous studies involving children, described in Fairclough et al., 2016 and van Hees et al., 2013 [37,39]. Weekdays with wear time of at least 10 h were included, and children with three or more valid days were included in the school week averages. This wear time inclusion criterion has previously been used in research exploring school day and segmented school day PA levels [41,42]. Published ENMO prediction equations were used to identify the cut-points for classifying activity as MVPA (3 METs (child-specific); AG cut-point = 201 mg, GA cut-point = 192 mg), as in Hildebrand et al., 2014 [43]. As there is no consensus as to the most appropriate ENMO ST cut-points [44], we also applied the Hildebrand et al., 2014 regression equations using 1.5 METs, which resulted in values of 51 mg (AG) and 61 mg (GA) [43]. As GA acceleration outputs are typically 9–11% higher than AG, we selected a comparable value of 50 mg for both devices as the cut-point to estimate ST.

### 2.4. Data Analysis

#### 2.4.1. Qualitative Data

Inductive thematic analysis allowed for themes to be identified and extracted from the child and teacher qualitative data driven by the aims of the research, as described by Braun and Clarke, 2006 [45]. Group interviews in particular were analyzed for individual accounts to explore acceptability and feasibility as opposed to consensus. Recently, pen profiles have been used to present data in similar PA qualitative research outputs [46,47,48]. This approach was adopted as an efficient illustration of key themes from the data. Diagrams which are similar to flow charts are created to present both examples of key verbatim quotes and also frequency data. As a result of this, it is deemed an appropriate and effective way of presenting data to researchers that have an affinity for both quantitative and qualitative approaches [47]. The pen profiles created were presented to two authors with previous experience in this analysis to ensure accuracy [47,48]. This also allowed for alternative interpretations with cross-examination in reverse, from pen profiles to transcripts. This process was repeated until an acceptable consensus had been reached (90% agreement level).

#### 2.4.2. Quantitative Data

A one-way analysis of variance (ANOVA) examined differences in the MVPA and ST across the whole school day between each intervention component. Accelerometer data were filtered using the ilevels parameters in GGIR to generate the MVPA and ST values during the teacher-defined intervention times as reported within the teachers’ recording sheets. Accelerometer data were also filtered for equivalent ‘usual practice’ classroom lessons, which occurred directly before or after the intervention period within the same school to act as comparison periods. The intervention time periods ranged from five to 15 min. To ensure all of the MVPA and ST accrued was included, 30-min windows were used to include the intervention components. These 30-min windows also helped to minimize any contamination of engagement in activity within the ‘usual practice’ windows. Children who had 10 h of wear time on the reported intervention days were included, regardless of whether they had three valid weekdays of 10-h wear time overall. Paired samples *t*-tests examined differences in the MVPA and ST between the intervention times and the usual-practice classroom times. This analytic approach was not suitable for use with the playground intervention data because there was no usual-practice comparator available. Instead, lunch-time playground data of schools participating in the playground intervention was compared to the other participating schools (who implemented the AB and BTM interventions). As these schools implemented class-time based interventions (ABs and BTM), the schools playground break time periods were not influenced by participation in the project. Teacher defined lunch playground times and independent *t*-tests were used to conduct the analysis. Statistical significance was set at 0.05 and effect sizes were represented by Cohen’s d. Analyses were conducted using the statistical package SPSS (v.23, SPSS Inc., Chicago, IL, USA).

## 3. Results

### 3.1. Qualitative Data

#### 3.1.1. Child Perceptions

**Figure 1:**
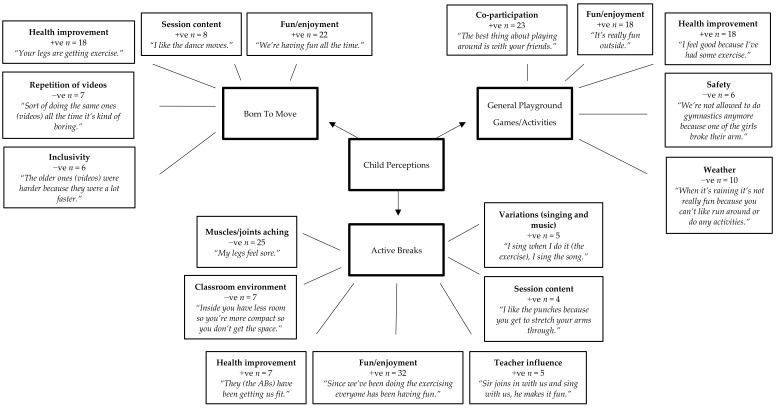
Children’s perceptions of each intervention. +ve = positive. −ve = negative.

Pen profiles representing the children’s perceptions are presented in Figure 1, with three higher order themes of each intervention (i.e., ABs, BTM, and playground). Positives (+ve; child reported likes) and negatives (−ve; child reported dislikes) of each intervention were the higher order sub-themes. There were nine sub-themes relating to ABs, which included, variations +ve (singing and music; *n* = 5), session content +ve (*n* = 4), health improvement +ve (*n* = 18), teacher influence +ve (*n* = 5), fun/enjoyment +ve (*n* = 32), muscle/joints aching −ve (*n* = 25), and the classroom environment −ve (*n* = 7). There were five sub themes of the BTM, which were health improvement +ve (*n* = 18), session content +ve (*n* = 8), fun/enjoyment +ve (*n* = 22), video repetition −ve (*n* = 7), and inclusivity −ve (*n* = 6). There were five sub themes for playground activities. However, these were in relation to general and traditional playground games/activities/sports and not the specific new teacher led games of the intervention. Sub themes included, co-participation +ve (*n* = 23), fun/enjoyment +ve (*n* = 18), health improvement +ve (*n* = 12), safety −ve (*n* = 6), and weather −ve (*n* = 10).

#### 3.1.2. Teacher Perceptions

**Figure 2:**
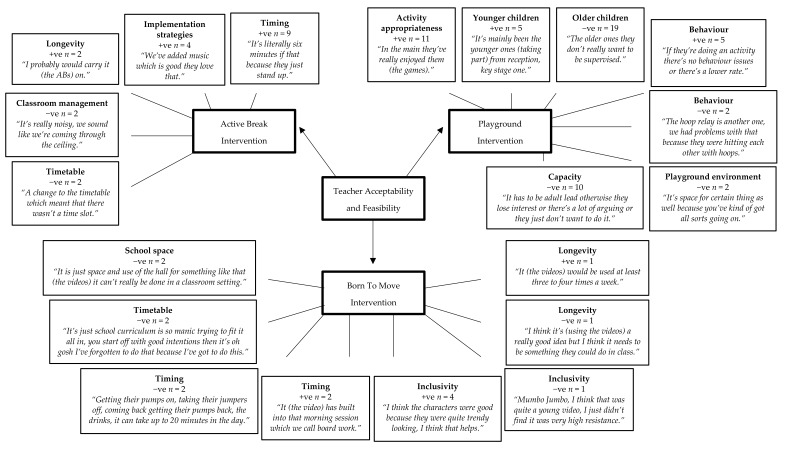
Teacher acceptability and feasibility of each intervention. +ve = positive. −ve = negative.

Pen profiles representing teacher acceptability and feasibility are presented in Figure 2, again with three higher order themes of each intervention. Positive sub-themes included useful methods for implementation and acceptability. Negative sub themes included barriers towards implementation. There were five AB sub-themes, which were longevity +ve (*n* = 3), implementation strategies +ve (*n* = 4), timing +ve (*n* = 9), timetable −ve (*n* = 2), and classroom management −ve (*n* = 2). Teachers who implemented BTM reported eight sub-themes of, timing +ve (*n* = 2), inclusivity +ve (*n* = 2), longevity +ve (*n* = 1), timing −ve (*n* = 2), inclusivity −ve (*n* = 2), longevity −ve (*n* = 1), school space −ve (*n* = 2), and timetable −ve (*n* = 2). When discussing the playground games that playground supervisory staff were asked to implement, seven sub-themes identified were, activity appropriateness +ve (*n* = 11), younger children +ve (*n* = 5), behavior +ve (*n* = 5), older children −ve (*n* = 19), behavior −ve (*n* = 2), playground environment −ve (*n* = 4), and capacity −ve (*n* = 10).

#### 3.1.3. Teacher Reported Implementation

Teachers typically implemented ABs once a day in the morning for five minutes. Some teachers reported implementing two ABs a day; however, this was less common (average = five days across the four-week implementation period). There were only three days across the three schools (one in each) that ABs were not implemented. BTM videos were implemented during mornings, afternoons, and just before the end of the school day. One school consistently implemented the videos every day, but implementation was infrequent in the other school. Playground supervisory staff typically implemented the playground active games with younger children (ages five to seven years).

### 3.2. Quantitative Data

One hundred and ninety-five children (87% compliance) wore an accelerometer for the defined wear time to establish the school day ST and MVPA levels (Table 1). There were significant differences in the whole school day MVPA levels between children who received the AB (32.3 min) and BTM (45.7 min; *p* < 0.001, d = −0.9), and the BTM and playground (37.0 min; *p* = 0.007, d = 0.6) interventions. Significant differences in the whole school day ST levels between children who received the AB (259.9 min) and BTM (237.0 min; *p* < 0.001, d = 0.7), and the AB and playground (232.9 min; *p* < 0.001, d = 1) interventions were also observed.

#### 3.2.1. Active Break Intervention

Twelve ABs were analyzed. The average ST and MVPA times during the 30-min windows, including ABs and the comparative ‘usual practice’ class time, are presented in Table 2. There were 4.8 min of MVPA accrued on average during the ABs, which was significantly higher than during the usual practice (*p* < 0.001, d = 2.2). ST during ABs was significantly lower (20.3 min) than during the usual practice lessons (25.3 min; *p* = 0.009, d = −1.0).

#### 3.2.2. Born to Move Intervention

Seven BTM video PA sessions were analyzed. ST and MVPA times during the 30-min windows, including the BTM videos and the comparative usual practice, are presented in Table 2. MVPA during the BTM videos (8.6 min) was significantly higher compared to the usual practice (1.8 min; *p* = 0.002, d = 2.1). ST during the BTM videos (12.5 min) was also significantly lower than during the usual practice (21.3 min; *p* = 0.003, d = −1.8).

#### 3.2.3. Playground Intervention

Lunchtime playground ST and MVPA are presented in Table 3. On average, the time on the playground at lunch was 37 min (range 25–45 min). Within the playground intervention schools, ST% was significantly lower (35.4%) in comparison to the other schools (43.8%, *p* < 0.001, d = −0.7). Lunchtime playground MVPA during the playground intervention schools was 17.2%, compared to 14.6% (*p* = 0.08, d = 0.3).

## 4. Discussion

The primary aim of this study was to explore the acceptability and feasibility of three four-week single component school-based PA interventions. All participating schools were able to implement the intervention components but reported a range of implementation challenges. These implementation challenges included space and the school environment, as well as the competing demands of teachers and other members of staff, such as timetable constraints and other responsibilities. The secondary aim was to examine the effectiveness of the interventions on increasing PA levels and reducing ST. The accelerometry data evidenced some positive effects particularly in regard to the AB and BTM interventions.

### 4.1. Acceptability and Feasibility

The teacher sub-themes within the results section were based on the type of intervention implemented and the individual schools were not considered. Therefore, there was some variance in between-school teacher responses, with similar sub-themes portrayed as both positive and negative. For example, there were positive and negative timing sub-themes to the BTM intervention. This highlighted school differences. All schools were different in the way they worked day-to-day, and whilst the current study explored acceptability and feasibility, it was important to remember that what worked in one school may not work in another. Educational, school-based research is highly influenced by context which differs significantly from school to school, such as personnel, teaching methods, budgets, leadership, and support [49].

When asked about new teacher-led playground activities or games, none of the children in the participating schools reported taking part. Teachers recalled that the activities and games could be implemented with younger children (ages five to seven years), but a number of barriers prevented involvement from older children (age groups of those who participated in the study). Recess focused PA research has studied age with inconclusive results [50]. Teacher-reported barriers included the older children not wanting supervision or structure. Despite the training received, teachers still found it challenging to engage students in activities. Previous research has reported similar issues, stating that teachers found it difficult to participate in playground activities whilst maintaining their responsibility to monitor the playground at the same time [51]. Teachers argued the need for activities for which children could engage in independently. Behavioral issues (e.g., arguments) or health and safety issues (e.g., administering first aid) commonly required teacher attention and prevented adult-led activities from being sustained. 

Enjoyment and health enhancements were themes from all the participating children across the different interventions. Enjoyment is deemed to be a crucial factor in health behavior change research of children, as it is a stable and consistent psychological construct which predicts PA participation and adherence [52,53,54]. Child enjoyment of integrating movement into classrooms has also been previously reported [55]. Thus, the children’s consistent and common reports of enjoyment for particularly the AB and BTM interventions increase the acceptability of these interventions for further use. Children reporting exercise participation and health enhancement provides a link to the predisposing factors described in the Youth PA Promotion Model [56]. Children in the current study recognized the perceived benefits of the additional PA to their day, which further reinforced the acceptability of the interventions.

A reported child-dislike of the ABs was lack of space which was also recognized by teachers. In other AB research, space has been reported as a consideration for implementation [57]. However, and again similar to the previous research [57], teachers in the current study did not report lack of space as something which would prevent them from implementing the ABs, but rather something they needed to consider and subsequently adapt the activity around. Teachers talked about positive adaptations and implementation strategies that they were able to use to ensure the ABs were compatible with the practice of participating classes. Strategies included incorporating learning and academic content, giving children extra challenges, participation with music playing, and allowing the children to choose the activity cards rather than this being a teacher decision. Previous research has suggested that when teachers see the positive attributes and outcomes of PA they adopt their own strategies which help movement and PA to be truly integrated into classroom life [58]. Integration is particularly important due to the well reported time constraints within schools [20]. Time in relation to the ABs was talked about positively by participating teachers, stating that the overall implementation and transitions to learning afterwards were quick. This was supportive of recent recommendations for practice, stating that classroom-based PA should have a minimum duration of 10 min [21]. However, unexpected changes to the timetable or particularly busy days could still prevent ABs from being implemented in the current study.

The longer duration of BTM videos (up to 20 min) was acknowledged by teachers as a barrier to implementation, this time period included getting to and from the hall/gym, as well as participation in the video. This was seen as a considerable amount of time to take away from a busy school curriculum. However, one teacher talked positively in relation to timing, stating that the videos had been integrated into and fitted well into the morning session. Previous research has reported goals and behavioral regulation to be facilitators of school-based PA, such as planning for and scheduling PA into the timetable [20]. A further barrier to the implementation of the BTM videos was the need for participation to take place in the school hall/gym. This area within most UK schools is used regularly for activities such as assemblies, PE lessons, and commonly doubles as a dining room at lunch time. It is likely that schools located in more affluent areas of the UK would also only have access to one school hall/gym and this environmental barrier is therefore unlikely to be specific to the two participating schools of the BTM intervention. This would support recently published feasible strategies for PA in schools, which stated that implementation should take place in the classroom [21]. This is primarily due to scheduling and timetabling issues in which access to the hall/gym cannot always be guaranteed at a time which suits the teacher [21]. Furthermore, more time is needed to get the children to and from the classrooms and halls/gyms.

### 4.2. Moderate to Vigorous Physical Activity and Sedentary Time

MVPA during the 30-min windows of the school day which included an AB was significantly higher than the comparable 30-min windows of ‘usual practice’ classroom learning. Findings from previous classroom AB research have also demonstrated the effectiveness of this approach for increasing PA levels. In a study of six schools, the minutes/day of ABs was positively associated with students’ MVPA, and the students were more likely to achieve the recommended 30 min/day of MVPA during school hours if their teachers reported implementing ABs [59]. There were 4.8 min of MVPA accrued on average during the 30-min windows of school time, which included participation in an AB. In comparison, there were 8.6 min of MVPA accrued on average during the 30-min windows, including participation in a BTM video. Whilst the videos were implemented for longer than the ABs (10 min compared to five minutes), they were also implemented in the school hall/gym. Although mentioned previously as a barrier towards implementation, children participating in the BTM videos were resultantly provided with increased space in comparison to the classroom environment. During the recess period of the school day, although within a different location of the outdoors compared to the school hall/gym, available play space per child has been found to predict increased vigorous PA and decreased ST [60]. Overall, MVPA was highest on average in the schools participating in the BTM intervention. Additional engagement in MVPA predicts positive effects with decreased child adiposity for example, and is therefore of significance for health [61,62]. However, this research was based upon on 15 min reallocations of time which was more than the amount which children engaged in, for example, during the ABs and BTM videos. Research relating more specifically to school hours only, and using 10 min reallocations of time, found that when ST or LPA were substituted with MVPA, there were favorable relationships with adiposity and cardiorespiratory fitness [63]. Therefore, the combination of strategies through multi-component interventions where children engage in various PA opportunities during the school day to achieve changes in MPVA levels, which are meaningful in terms of health benefits, is warranted.

In all participating schools, the percentage of time spent in MVPA on the playground was lower than the 30–35% figures previously reported through accelerometers, regardless of the intervention implemented [60]. Largely, previous school-based recess interventions have focused on changing the physical environment of playgrounds, for example, with markings and equipment [64,65]. More similar to the current intervention, previous studies have implemented playground age-appropriate games and activities. For example, in the ‘Recess Enhancement Program’ external play coaches visited schools twice a week and encouraged teachers to facilitate games in the coach’s absence [65]. Conversely, trained researchers have been used to implement structured recess games [66]. Whilst both studies found positive effects on the MVPA outcomes, the outcomes sustainability could be questioned [65,66]. To have an external qualified coach across a whole school year would be a costly addition for schools. More sustainable approaches with minimal or less financial impact are warranted to improve recess MVPA in the long-term. In terms of the percentage ST on the playground, this was significantly lower in the playground intervention schools in comparison to the other participating schools. Although it is difficult to speculate why this was, given the perceived lack of take-up by the target children discussed from the qualitative data.

### 4.3. Strengths and Limitations

A strength of the current study was the triangulation of multiple data sources. Collecting data from the perspective of participating children and teachers, in addition to accelerometer data provided robust evidence of each intervention’s acceptability and feasibility. This approach was consistent with the MRC’s guidance for developing and evaluating complex interventions, which advocates for the combined use of qualitative and quantitative methods when assessing feasibility [19]. Furthermore, the triangular consensus procedure of the qualitative data allowed for alternative perspectives to be presented and it ensured methodological rigor and credibility, whilst the comparison of pen profiles with verbatim data accentuated dependability. Self-select interventions at the school-level have been previously used in multi-component interventions, and have been successful at positively impacting the PA levels [14,16,67]. Within AS!BC, and Finnish Schools on the Move, the schools had the opportunity to plan interventions themselves through a ‘bottom-up’ choice-based approach. However, the use of self-select interventions may be viewed as a limitation of the current study as it may bias the observed results, with schools selecting interventions that they believed would be most enjoyable or successful with pupils. Additionally, this lack of random assignment to ‘treatments’ and lack of random selection of participants were limitations as these practices are said to be the most powerful means of controlling threats to internal and external validity [68]. Other limitations included the short implementation period of four weeks. A pragmatic approach was needed in the wider context of the AS:Sk project, where a staggered start four-week implementation period across the seven schools was most suitable. Owing to this short implementation phase, it was possible that the novelty of the interventions contributed to any favorable differences in PA outcomes. Additionally, the use of different accelerometer models (the AG and GA) was a limitation. Comparisons between the MVPA outcomes should be made with caution due to the technical differences between the accelerometers used, with GA values typically higher than AG, particularly for the MVPA [38]. Furthermore, as a result of missing recording sheets and unavailable ‘usual practice’ directly before or after, a limited number of AB (*n* = 12) and BTM (*n* = 7) intervention periods were extracted for analysis of the MVPA and ST data. Understanding the impact of the playground intervention on the PA outcomes was limited due to the lack of comparable usual practice, and the lack of a control group. Comparison of the playground periods of the school day between the playground intervention schools and the AB and BTM intervention schools was a limitation based upon convenience. In terms of the qualitative data, the allocation of children to groups by teachers was a limitation that may have caused bias within the groups. However, this teacher allocation was most convenient to ensure that all children participated within the given time available.

## 5. Conclusions

The AB intervention component was perceived to be feasible and acceptable, and it resulted in increased levels of MVPA among 9–10-year-old children during the school day. Teachers were able to implement ABs regularly and the children reported them enjoyable to take part in. BTM videos or similar high intensity instructional exercise videos, are less feasible to implement on a daily basis. Whilst the BTM intervention component also led to engagement in MVPA and children found the videos enjoyable, access to sufficient space for implementation was cited by teachers as a challenge. This type of intervention may be more feasible to implement on a less regular basis, for example, two-three times per week. Playground staff reported that they found it challenging to implement activities or games due to their competing role responsibilities. Staff also reported differences between the engagement of younger and older children and they perceived older children to prefer independence. Based on this feedback, games or activities which could be undertaken independently by children without the need for teacher initiation or support, whilst ongoing, may be more feasible to implement. Future research should explore the feasibility and acceptability of these example interventions when implemented simultaneously in a multi-component intervention, in which they may consequently have the greatest potential for impacting MVPA levels.

## Figures and Tables

**Table 1:** Whole school day ST and MVPA by intervention component trialed (mean and standard deviation).

	AB Intervention (*n* = 101)	BTM Intervention (*n* = 43)	Playground Intervention (*n* = 51)	AB vs. BTM	AB vs. PI	BTM vs. PI
ST (minutes)	259.9 (30.6)	237.0 (33.4)	232.9 (25.7)	*p* < 0.001 d = 0.7	*p* < 0.001 d = 1.0	*p* = 0.8 d = 0.1
MVPA (minutes)	32.3 (13.0)	45.7 (15.6)	37.0 (14.2)	*p* < 0.001 d = −0.9	*p* = 0.8 d = −0.3	*p* = 0.007 d = 0.6

Abbreviations: ST, sedentary time; MVPA, moderate to vigorous physical activity; AB, active break; BTM, Born To Move; PI, playground intervention; d, Cohen’s d.

**Table 2:** ST and MVPA accrued during the 30-min windows including an AB, BTM video, and usual classroom practice (mean and standard deviation).

	AB	Usual Practice Pre/Post AB	*p*	d	BTM	Usual Practice Pre/Post BTM	*p*	d
ST (minutes)	20.3 (5.4)	25.3 (4.4)	0.009	−1.0	12.5 (5.2)	21.3 (4.7)	0.003	−1.8
MVPA (minutes)	4.8 (2.5)	0.9 (1.1)	<0.001	2.2	8.6 (4.0)	1.8 (2.6)	0.002	2.1

Abbreviations: AB, active break; BTM, Born To Move; ST, sedentary time; MVPA, moderate to vigorous physical activity; d, Cohen’s d.

**Table 3:** Percentage ST and MVPA accrued during the lunch-time break of the schools participating in the playground intervention, and the other remaining schools (mean and standard deviation).

	Playground Intervention Schools (*n* = 2)	Other Schools (*n* = 5)	*p*	d
Playground %ST	35.4 (9.1)	43.8 (16.2)	<0.001	−0.7
Playground %MVPA	17.2 (7.5)	14.6 (9.5)	0.08	0.3

Abbreviations: ST, sedentary time; MVPA, moderate to vigorous physical activity; d, Cohen’s d.
